# Randomized Trial of General Strength and Conditioning versus Motor Control and Manual Therapy for Chronic Low Back Pain on Physical and Self-Report Outcomes

**DOI:** 10.3390/jcm9061726

**Published:** 2020-06-03

**Authors:** Scott D. Tagliaferri, Clint T. Miller, Jon J. Ford, Andrew J. Hahne, Luana C. Main, Timo Rantalainen, David A. Connell, Katherine J. Simson, Patrick J. Owen, Daniel L. Belavy

**Affiliations:** 1 Institute for Physical Activity and Nutrition, School of Exercise and Nutrition Sciences, Deakin University, Geelong, VIC 3220, Australia; scott.tagliaferri@deakin.edu.au (S.D.T.); c.miller@deakin.edu.au (C.T.M.); luana.main@deakin.edu.au (L.C.M.); timo.rantalainen@jyu.fi (T.R.); p.owen@deakin.edu.au (P.J.O.); 2 Advance HealthCare, 157 Scoresby Rd, Boronia, VIC 3155, Australia; jford@advancehealthcare.com.au; 3 Low Back Research Team, College of Science, Health & Engineering, La Trobe University, Bundoora, VIC 3083, Australia; a.hahne@latrobe.edu.au; 4 Gerontology Research Center and Faculty of Sport and Health Sciences, University of Jyväskylä, 40014 Jyväskylä, Finland; 5 Imaging@Olympic Park, AAMI Park, 60 Olympic Boulevard, Melbourne, VIC 3004, Australia; d.connell@iop.net.au; 6 School of Exercise and Nutrition Sciences, Deakin University, Geelong, VIC 3220, Australia; katie_simson@hotmail.com

**Keywords:** exercise, spine, physiotherapy, physical therapy, rehabilitation

## Abstract

Exercise and spinal manipulative therapy are commonly used for the treatment of chronic low back pain (CLBP) in Australia. Reduction in pain intensity is a common outcome; however, it is only one measure of intervention efficacy in clinical practice. Therefore, we evaluated the effectiveness of two common clinical interventions on physical and self-report measures in CLBP. Participants were randomized to a 6-month intervention of general strength and conditioning (GSC; *n* = 20; up to 52 sessions) or motor control exercise plus manual therapy (MCMT; *n* = 20; up to 12 sessions). Pain intensity was measured at baseline and fortnightly throughout the intervention. Trunk extension and flexion endurance, leg muscle strength and endurance, paraspinal muscle volume, cardio-respiratory fitness and self-report measures of kinesiophobia, disability and quality of life were assessed at baseline and 3- and 6-month follow-up. Pain intensity differed favoring MCMT between-groups at week 14 and 16 of treatment (both, *p* = 0.003), but not at 6-month follow-up. Both GSC (mean change (95%CI): −10.7 (−18.7, −2.8) mm; *p* = 0.008) and MCMT (−19.2 (−28.1, −10.3) mm; *p* < 0.001) had within-group reductions in pain intensity at six months, but did not achieve clinically meaningful thresholds (20mm) within- or between-group. At 6-month follow-up, GSC increased trunk extension (mean difference (95% CI): 81.8 (34.8, 128.8) s; *p* = 0.004) and flexion endurance (51.5 (20.5, 82.6) s; *p* = 0.004), as well as leg muscle strength (24.7 (3.4, 46.0) kg; *p* = 0.001) and endurance (9.1 (1.7, 16.4) reps; *p* = 0.015) compared to MCMT. GSC reduced disability (−5.7 (−11.2, −0.2) pts; *p* = 0.041) and kinesiophobia (−6.6 (−9.9, −3.2) pts; *p* < 0.001) compared to MCMT at 6-month follow-up. Multifidus volume increased within-group for GSC (*p* = 0.003), but not MCMT or between-groups. No other between-group changes were observed at six months. Overall, GSC improved trunk endurance, leg muscle strength and endurance, self-report disability and kinesiophobia compared to MCMT at six months. These results show that GSC may provide a more diverse range of treatment effects compared to MCMT.

## 1. Introduction

Low back pain (LBP) occurs in 40–85% of people at some point in their lives [1,2] and remains the leading cause of reduced function and years lived with disability worldwide [3,4]. Most recent estimates suggest that the direct and indirect costs of LBP in Australia were AUD 9.17 billion per year [5] and in excess of USD 100 billion per year in the United States [6]. Many individuals with back pain achieve recovery in the first six weeks [7]. However, persistent LBP (beyond six weeks) is highly prevalent, with up to 71% of people with acute LBP not fully recovered at 1 year [8]. Persistent LBP beyond 12 weeks is defined as chronic LBP (CLBP) [9,10], and accounts for the majority of the costs of the condition [11]. 

Pain intensity is a common and important clinical outcome measure used in people with CLBP [12]. Conservative approaches, such as exercise training [13] and spinal manipulative therapy [14], are as effective as surgery for reducing pain intensity, yet is more cost effective and has a lower risk of complications [15]. People with CLBP present as a heterogenous population which highlights the need to provide individualized treatment approaches [16]. Therefore, it is now recommended that treatment of CLBP should not solely focus on pain intensity [17]. We have already reported intervertebral disc outcomes from this trial, an important part of the ‘bio’ in biopsychosocial [18]. Muscular strength and endurance [19], paraspinal muscular atrophy [20], cardiorespiratory fitness [21], kinesiophobia [22] and quality of life [23] are additional clinical measures that could be targeted by treatment in individuals with CLBP. To adopt a more robust biopsychosocial and individualized approach, these outcomes should also be considered by clinicians when treating someone with CLBP [17]. 

General strength and conditioning [24,25] and motor control exercise with adjunct spinal manipulative therapy [26] are commonly implemented clinical modalities that have been extensively studied for pain intensity. However, less is known about how these interventions can impact additional outcomes in CLBP [13]. These interventions may have additional benefits that are worth exploring, as this could assist with clinical justification and improving treatment efficacy. Therefore, the aims were to assess the efficacy of general strength and conditioning (GSC) compared to motor control exercise and manual therapy (MCMT) for treating CLBP on pain intensity and a range of important clinical outcomes that could assist with treatment justification. 

## 2. Materials and Methods

We have previously reported the protocol of this randomized clinical trial (RCT) [27], and in this paper we cite some of the a priori declared secondary outcomes. The primary outcomes of the RCT have been published elsewhere [18]. In short, the primary outcomes of the trial focused on the anabolic mechanisms of exercise on the intervertebral disc, with no benefits found for intervertebral disc measures [18]. Participants were randomized to one of two groups: (1) GSC; participants completed a periodized GSC program under the supervision of an exercise physiologist with the assistance of student exercise physiologists (*n* = 20) or (2) MCMT; participants underwent motor control exercise and manual therapy with a qualified physiotherapist (*n* = 20). The study was approved by Deakin University Human Research Ethics Committee (project ID: 2015-191) and registered with the Australian New Zealand Clinical Trials Registry (ACTRN12615001270505). Participants provided medical clearance and written consent prior to study participation. 

### 2.1. Participants

Participants were recruited from the general public between the inner and eastern suburbs of Melbourne (Victoria, Australia). The distribution of print and web-based advertising included local businesses, medical/health centers, Deakin University staff and students and social media. Potential participants registered their interest through a study website. Applicants were screened by phone against inclusion and exclusion criteria to determine eligibility prior to attending an assessment session.

#### 2.1.1. Inclusion and Exclusion

Eligible participants were aged 25–45 years old with non-specific CLBP (>3 months) between the T12 vertebra and gluteal fold with pain of 2–8 on the numerical rating scale of 0–10. Exclusion criteria included previous or planned spinal surgery, traumatic spinal injury (e.g., fracture or car accident), cauda equina symptoms, known structural scoliosis, radiculopathy, or non-musculoskeletal causes of LBP. Other exclusion criteria included the inability to communicate in English, current LBP treatment (to isolate the effect of exercise), current compensable claim for their LBP, current pregnancy, considering pregnancy within the next six months, having given birth within last nine months, current smoker, current anaemia, body mass ≥120 kg, history of seizures, epilepsy, stroke or head injury, taking medications for mental illness, have metal implants unsuitable for magnetic resonance imaging (MRI), having had nuclear medicine performed in last three months or are unable to attend two 1-h training sessions per week for six months and three 1-h testing sessions plus additional MRI scans. Individuals undertaking more than 150 min per week of self-reported moderate-vigorous exercise (including any participation in structured sport or gym-based activities) were excluded to see if increases in exercise influenced the intervertebral disc outcomes of the wider study [18]. 

#### 2.1.2. Randomization

An off-site researcher who had no contact with participants’ complete randomization (using block randomization with random block lengths and stratification for gender) [27]. Concealed allocation was achieved by the offsite researcher allocating participants in accordance with the randomization schedule.

### 2.2. Interventions

#### 2.2.1. General Strength and Conditioning 

Participants in GSC underwent six months of supervised gym-based sessions. Sessions were supervised at the Deakin Clinical Exercise Centre or Burwood YMCA (Burwood, Victoria, Australia). In the first three months, participants attended two 1-h training sessions per week, followed by a self-selected one or two sessions per week for the second three months. Participants began each session with 20 min of aerobic conditioning of running or walking on a treadmill, starting at 65% and progressing to 85% of the heart rate maximum. Resistance training consisted of lift (e.g., squat, deadlift), push (e.g., chest press), pull (e.g., cable row), trunk extension (e.g., supine bridge) and flexion (e.g., curl-up) exercises with phases of muscular strength, hypertrophy and endurance (Supplementary Table S1). Progression was applied on a time contingent basis [28]. Pain neuroscience education regarding central hypersensitivity in chronic pain was provided to participants in the first session [29]. Participants were also instructed to complete 20–40 min of three weekly independent home-based aerobic training sessions at 65–85% of heart rate maximum during the 6-month protocol [30]. The mode of home training was walking or jogging. Furthermore, participants were instructed to complete 5–10 min of mental rehearsal tasks of movements associated with kinesiophobia in the first six weeks [31]. 

#### 2.2.2. Motor Control Exercise plus Manual Therapy

Participants in MCMT received 10 physiotherapy sessions of 30 min in the first three months and two 30-min sessions at any period in the second three months. Treatment took place at Advance HealthCare (Boronia, Victoria, Australia). Exercises targeted the transversus abdominis, multifidus and pelvic floor muscles plus postural correction to restore optimal motor control during non-weight-bearing activities [32]. Exercises and progressions followed previous protocols of motor control exercise [26,33]. Targeting activation of the deep core muscles during specific functional activities were only included as a progression if they formed part of the participants’ goals (e.g., walking). The progression of exercises was on a pain-contingent basis [28]. Spinal manipulative therapy followed key principles, including anterior–posterior and transverse mobilizations to lumbar spinal joints as well as soft tissues to the lumbo-pelvic region [34], with techniques and dosage based on the needs of the participant and determined at the discretion of the treating physiotherapist following clinical examination. To overcome any distress that may alter motor control (e.g., inability to relax the abdominal wall), participants were provided with basic cognitive-behavioral education to reassure the participant of the safety of motor control exercise. A home exercise program was provided to participants to complete each day between sessions, consisting of motor control, pelvic floor and postural education exercises. 

### 2.3. Outcomes

Testing sessions were completed at baseline, three and six months. In addition to this, participants were requested to complete online pain questionnaires every fortnight. 

#### 2.3.1. Physical Outcomes

Isometric trunk extension and isometric trunk flexion used assess local muscle endurance [35]. One-repetition maximum (1-RM) leg press and maximum repetitions at 70% 1-RM was used to evaluate lower body strength and local muscle endurance [27]. Cardio-respiratory fitness was determined using an individualized ramp protocol on a motorized treadmill. Full details are reported in the protocol paper [27].

#### 2.3.2. Paraspinal Muscle Size

**Figure 1:**
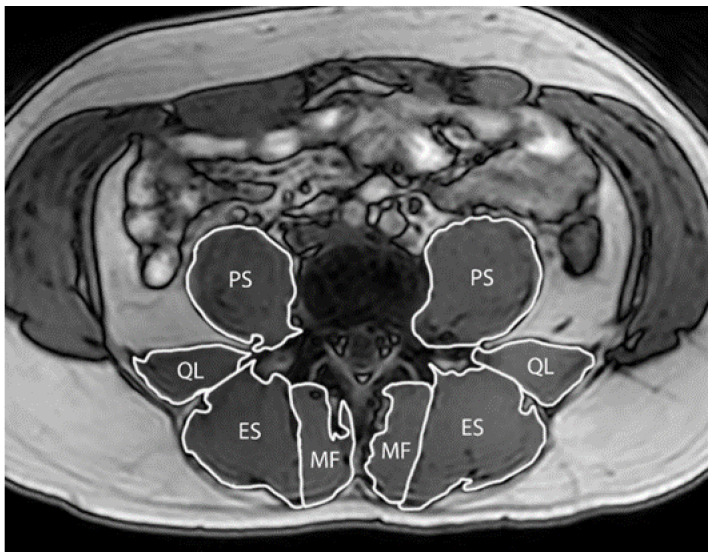
T2-weighted MRI image of the lumbar spine at the L4 vertebrae, showing traces of the multifidus (MF), erector spinae (ES), quadratus lumborum (QL) and psoas major (PS).

MRI was used to evaluate paraspinal muscle size (multifidus, psoas major, quadratus lumborum and erector spinae; Figure 1). The scanner operator was blinded to group allocation. Participants were asked to lay supine with a cushion wedged between both knees and hands placed above their head. A Phillips Ingenia 3.0 T scanner (Philips Healthcare, NSW, Australia) was used to collect 65 true-axial slices with a Dixon sequence (slice thickness: 3.5 mm, inter-slice distance: 0mm, repetition time: 3.6 ms, echo times: 1.21/2.3 ms, field-of-view: 250 AP × 300 RLmm interpolated to 432 × 432 pixels, bandwidth: 1526.3Hz) to encompass the paraspinal muscles from the sacrum up to and including T12. To blind the image analysis to allocation and study time-point, each dataset was assigned a random number prior to image analysis (obtained from www.random.org). ImageJ 1.50i (https://imagej.nih.gov/ij/) was used to trace around each muscle on the left and right sides. A custom ImageJ plugin (ROI Analyzer; https://github.com/tjrantal/RoiAnalyzer; https://sites.google.com/site/daniellbelavy/home/roianalyser) was then used to measure muscle area. Total volume (from first through to fifth vertebral level) and each lumbar level size was calculated. Data were averaged across left and right sides prior to further analysis.

#### 2.3.3. Self-Report Outcomes

The modified Oswestry disability index was used to measure self-reported disability due to LBP [36]. The questionnaire consists of 10 items, addressing various aspects of physical function. The Tampa scale of kinesiophobia is a 17-item questionnaire which assesses fear of (re)injury due to movement or activities [37]. Questions are scored on a 4-point Likert scale ranging from strongly disagree (one) to strongly agree (four). The 36-Item short-term health survey (SF-36) was used to assess quality of life [38]. The SF-36 assesses health over the previous four weeks in eight domains, which is then weighted to construct the physical and mental component summary scores [39]. Average pain intensity of the low back across the prior week was measured by the 0-100mm VAS [40]. A link to an online database (Qualtrics, Seattle, Washington, USA) for the pain questionnaire was sent fortnightly via email to the participant. 

### 2.4. Statistical Analysis

The original sample size calculation was based on primary intervertebral disc outcomes [27]. All statistical analyses were conducted using Stata/SE version 15 (StataCorp, College Station, TX, USA). Normality of distribution and equality of variance were assessed using Shapiro–Wilk’s test and Levene’s test, respectively. Non-normally distributed data underwent natural log transformation, but all data presented were derived from raw data. Independent *t*-tests were used to determine within-group changes. Linear mixed models with random effects accounting for the heterogeneity of variance according to study date and an intent-to-treat approach were used to determine between-group effects [41]. Missing data were dealt with using a maximum likelihood estimation within linear mixed models, satisfying intent-to-treat principles [42]. Significance levels of *p* < 0.05 were used for all statistical tests. 

## 3. Results

### 3.1. Descriptive Characteristics

**Figure 2:**
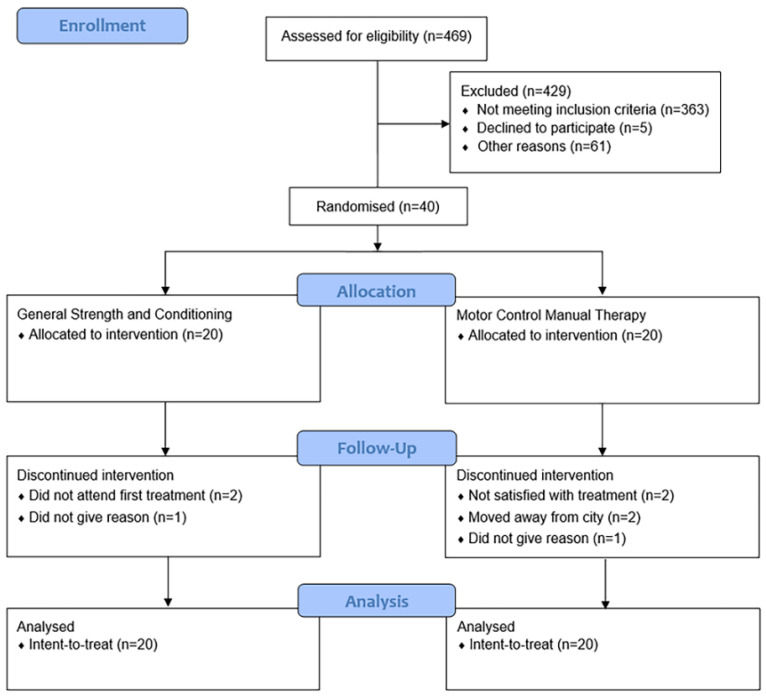
CONSORT diagram.

Descriptive characteristics of participants at baseline are presented in Table 1. Eight (20%) participants of the 40 (MCMT, *n* = 5; GSC, *n* = 3) dropped out over the 6-month intervention (Figure 2). The MCMT group attended a mean of 9/12 (77%) sessions, while GSC completed a mean of 31/52 (60%) available sessions. No serious adverse events occurred.

### 3.2. Physical Outcomes

Table 2 represents the changes in functional outcomes from baseline to three and six months. Trunk extension endurance increases were greater in GSC than MCMT at both three (mean (SD) difference, 105 (92)%; *p* = 0.007) and six months (88 (27)%; *p* = 0.004). Greater increases in trunk flexion endurance were seen in GSC at three (94 (39)%; *p* = 0.034) and six months (121 (39)%; *p* = 0.004). No difference in 1-RM leg press between groups was seen at three months, but increases were greater in GSC at six months (69 (37)%; *p* = 0.023). Leg endurance favored GSC at three (140 (62)%; *p* = 0.019) and six months (85 (63)%; *p* = 0.015). Cardio-respiratory fitness favored GSC (105 (3)%; *p* = 0.025) at three months only. 

### 3.3. Self-Reported Outcomes 

**Figure 3:**
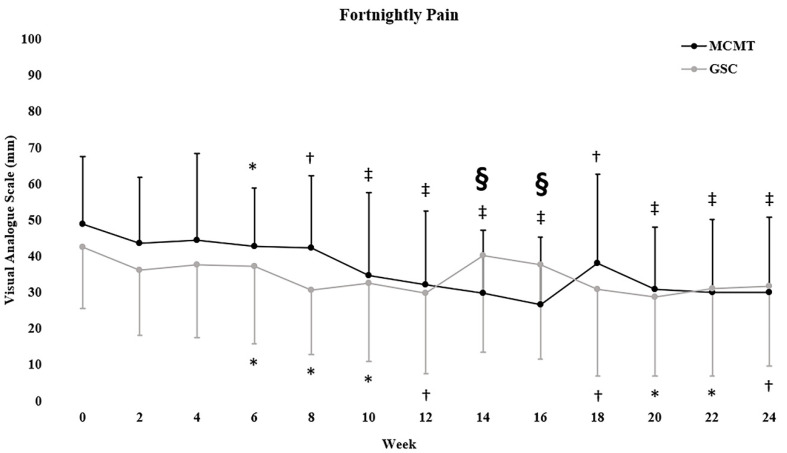
Mean ± standard deviation fortnightly pain (VAS) data. § *p* < 0.01 indicates significant between-group effect at that week. * *p* < 0.05, † *p* < 0.01, ‡ *p <* 0.001 indicate within-group change. Symbols above error bars refer to significant changes within the MCMT group, symbols below the error bars to the GSC group.

Reductions in pain intensity favored MCMT at week 14 and 16 (both, *p* = 0.003) only (Figure 3). Within-group changes in pain intensity were observed for both MCMT (*p* < 0.001) and GSC (*p* = 0.008) at six months. Table 3 shows the changes in other self-reported outcomes. Each group had reductions in the Oswestry disability index at both follow-ups, which were greater in GSC at three (86 (12)%; *p* < 0.015) and six months (54 (5)%; *p* = 0.041). GSC had greater improvements in the Tampa scale of kinesiophobia compared to MCMT at three (175 (33)%; *p* < 0.001) and six months (152 (33)%; *p* < 0.001).

### 3.4. Paraspinal Muscle Size 

Multifidus volume did not differ between groups at three or six months (Table 4). Within-group increases in multifidus volume were observed for GSC at six months only (*p* = 0.003). A between-group difference was seen for L5 multifidus size, favoring GSC at three months only (174 (2)%; *p* = 0.035, Supplementary Table S2). Within-group increases in multifidus size for GSC were seen at six months at L3 (*p* = 0.008), L4 (*p* = 0.013) and L5 (*p* = 0.001) vertebral levels, but not MCMT. Erector spinae, psoas major and quadratus lumborum size before and after the intervention at each lumbar vertebral level are displayed in Supplementary Tables S3–S5. 

## 4. Discussion

The main findings of this RCT were that both groups had significant reductions in pain intensity in individuals with CLBP after six months, however the results did not reach clinically meaningful within- or between-group thresholds. GSC also led to improved functional measures of trunk muscle endurance and leg muscle strength and endurance when compared to MCMT. Additionally, improvements in self-reported disability and kinesiophobia were greater following GSC than MCMT at six months.

### 4.1. Physical Outcomes

Trunk extension endurance improved in both groups at six months with between-group measures favoring GSC. Kell et al. [24] showed that 16 weeks of resistance training was superior to aerobic training and a usual care control for improving trunk extension endurance. Javadian et al. [43] showed that the addition of motor control training to an 8-week general exercise program did not lead to greater improvements in trunk endurance. Motor control exercise targets deep paraspinal muscles over global trunk muscles [32]. Specificity (e.g., training for a specific adaptation) is an important training variable with GSC training movements of trunk extension and flexion, and may reflect the results seen in our study [44]. Thus, if improving trunk endurance is a goal of treatment, further benefits can be achieved through GSC compared to MCMT.

Measures of leg muscle strength and endurance significantly favored GSC compared to MCMT at six months. Two prior RCTs of progressive resistance training in CLBP have assessed leg muscle strength with similar results to our study [25,45]. For motor control exercise, Aasa et al. [46] assessed isometric leg strength and compared this to a high-load deadlift exercise, with no difference seen between interventions. The differences observed between Aasa et al. [46] may exist due to the greater volume and frequency of training undertaken in the GSC group in our study [47]. A meta-analysis showed that loads of >60% 1-RM improve maximal strength more than training loads ≤60% 1-RM [48]. MCMT implemented a low-load exercise intervention (~30% effort) focusing on spinal musculature and was not expected to lead to strength gains in the lower body directly [33]. GSC may be used for additional benefits in improving muscular leg strength when compared to MCMT. 

No differences were seen in cardio-respiratory fitness between groups at the end of the intervention. Supporting our results, Chan et al. [49] showed no improvement in cardio-respiratory fitness over an 8-week period. However, Kell et al. [24] showed significant improvements in maximal oxygen consumption following supervised and periodized aerobic training. Compared to Chan et al [49], the participants in Kell et al. [24] had cardio-respiratory fitness levels below age-matched normative data [50]. Despite targeting a population not meeting the minimum daily physical activity recommendations, our participants did not have reduced cardio-respiratory fitness levels compared to age-matched norms [50]. Furthermore, increased supervision may have impacted the significant change, favoring GSC at three months, when supervision was at its highest, and therefore the stimulus of aerobic training may have been insufficient after three months [51]. Assessing cardio-respiratory fitness should help to determine a sufficient dose of aerobic exercise for individuals with CLBP. 

### 4.2. Self-Reported Outcomes

Both groups had reductions in pain intensity, however no differences were seen between groups at six months. There were between-group differences in pain intensity at week 14 and 16, with increases observed for GSC. This phase for GSC consisted of greater time under tension and eccentric contraction duration, therefore it is possible that increases in pain intensity could be related to delayed-onset muscle soreness [52,53]. Furthermore, within-group changes in pain intensity remained below clinically meaningful thresholds of 20/100 mm of the VAS (GSC mean change, −11 mm; MCMT mean change, −19 mm) [54]. The results of the current study support that pain intensity should not be the sole outcome to differentiate treatment efficacy. 

GSC showed greater reductions in self-reported disability compared to MCMT at both follow-ups. The within-group change was only clinically meaningful for the GSC group (defined as <10/100 point change on the Oswestry Disability index) but did not reach this threshold between-groups [55]. A previous meta-analysis showed motor control exercise to be superior to general exercise for improving self-reported disability [56]. However, previous RCTs [24,25,45] of progressive resistance training were not included in the meta-analysis [56]. Given the moderate correlation between kinesiophobia and disability, it is possible that the reductions in kinesiophobia in GSC were enough to further reduce perceptions of disability [57]. Our results show both MCMT and GSC can reduce self-reported disability, however greater benefits exist with GSC at three and six months.

GSC significantly improved kinesiophobia compared to MCMT in our study. Previous research by Moticone et al. [58] demonstrated that motor control exercise alone did not improve kinesiophobia when compared to motor control exercise plus cognitive behavioral therapy. Therefore, it is likely that the cognitive behavioral therapy resulted in improved kinesiophobia [58]. Cognitive behavioral therapy aims to identify and modify harmful cognitive behaviors (e.g., fear-avoidance and pain catastrophizing) in those with CLBP [59]. In the MCMT group in our study, cognitive behavioral therapy was only targeted at the safety of motor control exercise and may have not been adequate to alter kinesiophobia. However, mental rehearsal of feared movements and pain education in conjunction with exercise in GSC may have modified negative cognitions towards LBP, and subsequently reduced kinesiophobia [59]. Results from our study show that GSC has benefits for reducing kinesiophobia compared to MCMT.

### 4.3. Paraspinal Muscle Size 

No between-group differences were seen in any MRI outcomes at six-months, however a significant within-group change in multifidus volume was observed for GSC. Danneels et al. [60] similarly showed that motor control exercise did not improve lumbar multifidus size. However, the addition of dynamic-static trunk resistance training led to significant increases [60]. Contrastingly, Chung et al. [61] showed that motor control exercise increased lumbar multifidus size, although Chung et al. [61] only used a per-protocol analysis, which may overestimate the magnitude of change [62]. Berglund et al. [63] provided evidence that exercise may increase multifidus size, but was not conclusive regarding which type of exercise may be more beneficial. Generally, loads of 40–80%1-RM are recommended to maximize muscular hypertrophy [48]. Motor control exercise recruits the multifidus at less than 30% 1-RM, which is unlikely to promote muscular hypertrophy [33]. Our results support the notion that resistance training at loads of 40–80% 1-RM may be required to increase multifidus size.

### 4.4. Strengths and Limitations

This study was strengthened by an offsite researcher with no participant contact conducting randomization. MRI data was processed and analyzed by a blinded assessor. Finally, an experienced therapist oversaw each of the two interventions to ensure protocol adherence and consistency. 

For limitations, five participants dropped out from MCMT (25%), while three dropped out from GSC (15%). To minimize the risk of bias due to attrition, a full case of intent-to-treat analysis was completed [64]. There were differences between groups in the number of sessions and face-to-face time with clinicians, however, this was a pragmatic approach, reflective of current clinical practice [65]. Additionally, most of the improvements were already favorable towards GSC at three months. Therefore, the total number of sessions needed to achieve differences in these outcomes may not be as large as the maximum number of sessions in our study. We requested that participants complete an exercise diary for their home program, however, due to poor adherence and reporting, we were unable to provide details on their adherence. Given that we screened 469 individuals with only 40 eventually included in the trial, these results may only be generalizable to a smaller population of individuals with CLBP who fit within our inclusion and exclusion criteria [66]. Lastly, given that the primary aim of the trial was targeted at anabolism of the intervertebral disc, within this sample, we did not consider whether there were specific sub-groups that may benefit from particular treatments [67]. For example, in the MCMT group, not all participants presented with hypomobility that could benefit from spinal manipulative therapy, meaning that individuals may have not been best matched to this treatment approach [26]. Future trials may want to assess whether sub-groups can have additional clinical benefits from matched treatments to maximize intervention efficacy [67].

## 5. Conclusions

In our study, both MCMT and GSC had reductions in pain intensity, however the results did not reach clinically meaningful within- or between-group thresholds. GSC produced significantly greater improvements in trunk endurance, leg muscle strength and endurance, self-reported disability and kinesiophobia compared to MCMT. Therefore, GSC may achieve a more diverse range of treatment effects than MCMT and should be considered by clinicians when these are important outcomes to be improved for their patient. These results highlight the additional clinical benefits of GSC when compared to MCMT, when treatment of CLBP is sought by patients or endorsed by clinicians. 

## Figures and Tables

**Table 1:** Descriptive characteristics of participants at baseline of those randomized to general strength and conditioning (GSC) and motor control manual therapy (MCMT).

	GSC (*n* = 20)	MCMT (*n* = 20)
**Age, years**	34.8 (4.9)	34.6 (7.2)
**Male, *n* (%)** **Female, *n* (%)**	11 (55) 9 (45)	10 (50) 10 (50)
**Height, cm**	169.6 (7.7)	172.5 (9.1)
**Weight, kg**	77.8 (13.5)	76.9 (16.8)
**Body Mass Index, (kg/m^2^)**	27.1 (4.9)	25.4 (4.2)
**Medication, *n* (%) ^b^**	0 (0)	1 (5)
**Low Back Pain (0–100) ^c^**	41.4 (17.8)	48.9 (19.1)

Values are displayed as mean (standard deviation) unless specified. ^b^ Reported medication at baseline included any analgesic medication (*n* = 1, paracetamol/codeine). ^c^ Average low back pain intensity over the prior week measured by the visual analogue scale.

**Table 2:** Intent-to-treat analysis using linear mixed models on physical outcomes at baseline, three and six months in participants randomized to general strength and conditioning (GSC) and motor control manual therapy (MCMT).

	Baseline Values and Within-Group Changes					
	GSC		MCMT		GSC vs. MCMT	
	Mean (SD) Mean (95% CI)	*p*-Value	Mean (SD) Mean (95% CI)	*p*-Value	Net Difference (95% CI)	Group × Time
Trunk Extension Endurance (s) ^*^						
Baseline	101.9 (58.2)	**<0.001**	88.8 (35.9)	0.305	51.5 (4.5, 98.4)	**0.007**
∆ 3 months	74.8 (38.8, 110.8)		23.3 (−6.0, 52.6)			
∆ 6 months	133.7 (97.7, 169.7)	**<0.001**	51.8 (22.5, 81.1)	**<0.001**	81.8 (34.8, 128.8)	**0.004**
Trunk Flexion Endurance (s) ^*^						
Baseline	81.4 (71.5)	**0.001**	60.4 (40.5)	0.864	19.3 (−11.7, 50.5)	**0.034**
∆ 3 months	30.4 (5.6, 55.1)		11.0 (−6.7, 28.7)			
∆ 6 months	68.4 (43.6, 93.2)	**<0.001**	16.8 (−0.9, 34.5)	0.053	51.5 (20.5, 82.6)	**0.004**
**1-RM Leg Press (kg)**						
Baseline	141.1 (44.2)	**<0.001**	130.4 (43.6)	**0.040**	16.6 (−4.5, 37.7)	0.123
∆ 3 months	29.6 (13.0, 46.2)		13.1 (0.6, 25.5)			
∆ 6 months	47.7 (30.7, 64.7)	**<0.001**	23.1 (10.6, 35.5)	**<0.001**	24.7 (3.4, 46.0)	**0.023**
**Leg Press Endurance (repetitions)**						
Baseline	18.6 (5.4)	**0.001**	21.9 (5.8)	0.280	8.7 (1.4, 15.9)	**0.019**
∆ 3 months	10.7 (4.6, 16.9)		1.9 (−1.5, 5.4)			
∆ 6 months	15.4 (9.1, 21.7)	**<0.001**	6.2 (2.7, 9.7)	**0.001**	9.1 (1.7, 16.4)	**0.015**
**Peak Oxygen Consumption (mL/kg/min)**						
Baseline	38.0 (8.4)	**0.027**	38.8 (6.7)	0.333	3.2 (0.4, 6.0)	**0.025**
∆ 3 months	2.2 (0.2, 4.1)		−1.0 (−3.0, 1.0)			
∆ 6 months	2.4 (0.4, 4.3)	**0.019**	1.1 (−1.0, 3.2)	0.295	1.3 (−1.6, 4.2)	0.380

Data are: baseline unadjusted mean ± standard deviation (SD); within-group unadjusted mean absolute change with 95% confidence intervals (CI); net difference (95% CI) were calculated by subtracting unadjusted within-group absolute changes from baseline to three and six months for MCMT and GSC. * Analysis used natural log-transformed data.

**Table 3:** Intent-to-treat analysis using linear mixed models on self-reported outcomes at baseline, three and six months in participants randomized to general strength and conditioning (GSC) and motor control manual therapy (MCMT).

	Baseline Values and Within-Group Changes					
	GSC		MCMT		GSC vs MCMT	
	Mean (SD) Mean (95% CI)	*p*-Value	Mean (SD) Mean (95% CI)	*p*-Value	Net Difference (95% CI)	Group × Time
**Oswestry Disability Index (0–100)**						
Baseline	24.5 (12.1)	**<0.001** **<0.001**	23.4 (8.5)	**0.031** **<0.001**	−6.7 (−12.2, −1.3) −5.7 (−11.2, −0.2)	**0.015** **0.041**
∆ 3 months	−11.3 (−15.0, −7.6)		−4.5 (−8.5, −0.4)			
∆ 6 months	−13.5 (−17.2, −9.8)		−7.7 (−11.8, −3.5)			
**Tampa Scale of Kinesiophobia (17–68)**						
Baseline	38.7 (5.6)	**<0.001** **<0.001**	38.6 (6.0)	0.658 0.321	−6.7 (−10.0, −3.4) −6.6 (−9.9, −3.2)	**<0.001** **<0.001**
∆ 3 months	−6.0 (−8.7, −3.4)		0.4 (−1.5, 2.4)			
∆ 6 months	−7.4 (−10.0, −4.7)		−1.0 (−3.0, 1.0)			
**SF-36 Physical Health (0–100)**						
Baseline	42.6 (8.4)	**<0.001** **<0.001**	41.2 (9.6)	**0.001** **<0.001**	−0.7 (−6.7, 5.3) 0.6 (−5.5, 6.7)	0.813 0.843
∆ 3 months	7.5 (3.7, 11.3)		8.2 (3.5, 12.9)			
∆ 6 months	10.4 (6.6, 14.2)		9.7 (4.9, 14.5)			
**SF-36 Mental Health (0–100)**						
Baseline	31.5 (12.4)	0.098 **0.001**	33.4 (13.9)	0.959 0.106	7.1 (−3.8, 18.0) 7.5 (−3.5, 18.6)	0.202 0.179
∆ 3 months	6.8 (−1.2, 14.7)		−0.2 (−7.6, 7.2)			
∆ 6 months	13.7 (5.7, 21.6)		6.2 (−1.3, 13.7)			

Data are: baseline unadjusted mean ± standard deviation (SD); within-group unadjusted mean absolute change with 95% confidence intervals (CI); net difference (95% CI) were calculated by subtracting unadjusted within-group absolute changes from baseline to three and six months for MCMT and GSC.

**Table 4:** Intent-to-treat analysis using linear mixed models on average total of multifidus, erector spinae, psoas major and quadratus lumborum volume at baseline, three and six months in participants randomized to motor control manual therapy (MCMT) and general strength and conditioning (GSC).

	Baseline Values and Within-Group Changes					
	GSC		MCMT		GSC vs. MCMT	
	Mean (SD) Mean (95% CI)	*p*-Value	Mean (SD) Mean (95% CI)	*p*-Value	Net Difference (95% CI)	Group × Time
**Multifidus Volume (cm^3^)**						
Baseline	18.2 (4.0)	0.102	18.0 (5.5)	0.477	0.6 (−0.1, 1.4)	0.096
∆ 3 months	0.4 (−0.1, 1.0)		−0.2 (−0.7, 0.3)			
∆ 6 months	0.8 (0.3, 1.3)	**0.003**	0.2 (−0.3, 0.7)	0.463	0.6 (−0.1, 1.4)	0.116
**Erector Spinae Volume (cm^3^)**						
Baseline	45.9 (11.2)	0.506	48.7 (15.3)	0.884	0.5 (−1.2, 2.2)	0.570
∆ 3 months	0.4 (−0.8, 1.6)		−0.1 (−1.3, 1.1)			
∆ 6 months	0.1 (−1.1, 1.3)	0.810	0.1 (−1.1, 1.4)	0.822	0.0 (−1.7, 1.7)	0.998
**Psoas Major Volume (cm^3^)**						
Baseline	32.8 (9.7)	0.697	32.5 (11.5)	0.157	0.3 (−1.1, 1.7)	0.677
∆ 3 months	−0.2 (−1.4, 0.9)		−0.5 (−1.3, 0.2)			
∆ 6 months	0.2 (−0.9, 1.4)	0.686	0.0 (−0.7, 0.8)	0.967	0.2 (−1.2, 1.7)	0.761
**Quadratus Lumborum Volume (cm^3^)**						
Baseline	11.2 (3.4)	0.428	11.6 (4.4)	0.697	0.0 (−0.8, 0.9)	0.933
∆ 3 months	0.2 (−0.3, 0.6)		0.1 (−0.6, 0.9)			
∆ 6 months	0.1 (−0.4, 0.5)	0.813	0.3 (−0.5, 1.0)	0.472	−0.2 (−1.0, 0.6)	0.614

Data are: baseline unadjusted mean ± standard deviation (SD); within-group unadjusted mean absolute change with 95% confidence interval (CI); net difference (95% CI) were calculated by subtracting unadjusted within-group absolute changes from baseline to three and six months for MCMT and GSC.

## Supplementary Materials

The following are available online at https://www.mdpi.com/2077-0383/9/6/1726/s1. Table S1: Periodization model for the 6-month general strength and conditioning (GSC) intervention. Table S2: MRI outcomes for multifidus size of the middle three slices of each spinal level (L1-L5) at baseline, three and six months in participants randomized to motor control manual therapy (MCMT) and general strength and conditioning (GSC). Table S3: MRI outcomes for erector spinae size of the middle three slices of each spinal level (L1-L5) at baseline, three and six months in participants randomized to motor control manual therapy (MCMT) and general strength and conditioning (GSC). Table S4: MRI outcomes for psoas major size of the middle three slices of each spinal level (L1-L5) at baseline, three and six months in participants randomized to motor control manual therapy (MCMT) and general strength and conditioning (GSC). Table S5: MRI outcomes for quadratus lumborum size of the middle three slices of each spinal level (L1-L5) at baseline, three and six months in participants randomized to motor control manual therapy (MCMT) and general strength and conditioning (GSC).

## Trial Registration

Australian New Zealand Clinical Trials Registry (ACTRN12615001270505).
