# Supplementary material for: Randomized Trial of General Strength and Conditioning versus Motor Control and Manual Therapy for Chronic Low Back Pain on Physical and Self-Report Outcomes
Source: J Clin Med. 2020 Jun 3;9(6):1726. doi: 10.3390/jcm9061726 (PMC7355598; doi:10.3390/jcm9061726)
Supplement: Supplementary file 1 [file jcm-09-01726-s001.pdf]

TABLE S1. Periodization model for the 6-month general strength and conditioning (GSC) intervention.

Week	Goal	Intensity	Frequency	Routine	Time under tension	Progression
**1-4**	Familiarization, motor control and local muscular endurance	12-15 reps performed at 2 reps below volitional fatigue x 2 sets, 1 min rest between sets.	2/wk	Full body	2 sec concentric, 2 sec eccentric	Once completion of 2 sets of 15 reps at 2 consecutive training sessions resistance is increased
**5-10**	Muscular strength	6-10 reps performed at 2 reps below volitional fatigue x 2-3 sets, 2 min rest between sets	2/wk	Full body	2 sec concentric, 2 sec eccentric	Once completion of 2 sets of 10 reps at 2 consecutive training sessions workload increase to 3 sets. Then progression made through increased resistance.
**11**	De-load	10 reps at 80% of resistance used in the previous week x 3 sets, 2 min rest between sets	2/wk	Full body	2 sec concentric, 2 sec eccentric	Nil
**12-15**	Local muscular endurance	12-15 reps performed at 2 reps below volitional fatigue x 3 sets, 1 min rest between sets.	1-2/wk	Full body	5 sec concentric, 5 sec eccentric	Once completion of 3 sets of 15 reps at 2 consecutive training sessions resistance is increased
**16-19**	Muscular strength	6-10 reps performed at 2 reps below volitional fatigue x 3-4 sets, 2 min rest between sets	1-2/wk	Full body	2 sec concentric, 2 sec eccentric	Once completion of 3 sets of 10 reps at 2 consecutive training sessions workload increase to 4 sets. Then progression made through increased resistance.
**20-25**	Local muscular endurance	20-25 reps performed at 2 reps below volitional fatigue x 3 sets, 1 min rest between sets.	1-2/wk	Full body	1-2 sec concentric, 1-2 sec eccentric	Once completion of 3 sets of 25 reps at 2 consecutive training sessions resistance is increased

wk = week, reps = repetitions, sec = seconds, min = minutes.

TABLE S2. MRI outcomes for multifidus size of the middle three slices of each spinal level (L1-L5) at baseline, three and six months in participants randomized to general strength and conditioning (GSC) and motor control manual therapy (MCMT).

	**Baseline values and within group changes**				**GSC vs MCMT**	
	**GSC**		**MCMT**			
	**Mean (SD)<br>Mean (95% CI)**	**P-value**	**Mean (SD)<br>Mean (95% CI)**	**P-value**	**Net difference (95% CI)**	**Group x Time**
**L1 Multifidus Size (mm²)**						
Baseline	234.5 (75.2)		235.8 (73.1)			
Δ 3 months	9.7 (-2.3, 21.6)	0.112	-10.2 (-18.8, -1.6)	**0.019**	19.9 (5.0, 34.8)	**0.009**
Δ 6 months	6.5 (-5.5, 18.5)	0.287	-2.8 (-11.6, 6.0)	0.529	9.3 (-5.7, 24.4)	0.225
**L2 Multifidus Size (mm²)**						
Baseline	318.1 (100.0)		313.4 (106.8)			
Δ 3 months	12.2 (-8.6, 33.0)	0.251	-10.0 (-21.3, 1.0)	0.052	23.1 (-0.8, 47.1)	0.058
Δ 6 months	3.8 (-17.0, 24.6)	0.721	-9.0 (-20.0, 2.0)	0.109	13.2 (-11.1, 37.4)	0.287
**L3 Multifidus Size (mm²)**						
Baseline	480.0 (112.3)		455.6 (173.8)			
Δ 3 months	9.9 (-9.6, 29.5)	0.318	-9.1 (-37.0, 18.8)	0.521	19.1 (-14.5, 52.8)	0.266
Δ 6 months	26.3 (6.7, 45.8)	**0.008**	-0.0 (-28.6, 28.6)	0.999	26.3 (-7.8, 60.4)	0.130
**L4 Multifidus Size (mm²)**						
Baseline	739.3 (141.3)		742.7 (245.0)			
Δ 3 months	23.7 (-6.1, 53.5)	0.119	-13.9 (-47.3, 19.6)	0.416	37.6 (-7.1, 82.3)	0.099
Δ 6 months	37.8 (8.0, 67.6)	**0.013**	-5.8 (-40.1, 28.5)	0.741	43.6 (-1.7, 88.9)	0.059
**L5 Multifidus Size (mm²)**						
Baseline	781.4 (160.3)		790.5 (192.5)			
Δ 3 months	37.7 (11.2, 64.2)	**0.005**	-3.0 (-29.7, 23.9)	0.831	40.6 (2.9, 78.3)	**0.035**
Δ 6 months	46.0 (19.5, 72.5)	**0.001**	13.0 (-14.5, 40.4)	0.355	33.0 (-5.1, 71.2)	0.090

Data are: baseline unadjusted mean ± standard deviation (SD); within-group unadjusted mean absolute change with 95% confidence interval (CI); net difference (95% CI) were calculated by subtracting unadjusted within-group absolute changes from baseline to 3-and 6-months for MCMT and GSC.

TABLE S3. MRI outcomes for erector spinae size of the middle three slices of each spinal level (L1-L5) at baseline, three and six months in participants randomized to general strength and conditioning (GSC) and motor control manual therapy (MCMT).

	Baseline values and within group changes				GSC vs MCMT	
	GSC		MCMT			
	Mean (SD)<br>Mean (95% CI)	P-value	Mean (SD)<br>Mean (95% CI)	P-value	Net difference (95% CI)	Group x Time
**L1 Erector Spinae Size (mm^2)**						
Baseline	1630.0 (328.2)		1695.4 (571.2)			
Δ 3 months	23.7 (-33.1, 80.4)	0.413	-13.2 (-72.6, -46.2)	0.663	36.9 (-45.3, 119.2)	0.378
Δ 6 months	53.7 (-3.0, 110.5)	0.063	-2.4 (-63.3, 58.5)	0.938	56.1 (-27.2, 139.3)	0.187
**L2 Erector Spinae Size (mm^2)**						
Baseline	1806.7 (410.7)		1847.8 (585.3)			
Δ 3 months	24.1 (-34.3, 82.6)	0.419	8.3 (-36.8, 53.4)	0.719	15.9 (-58.8, 90.7)	0.676
Δ 6 months	43.3 (-15.2, 101.8)	0.147	15.3 (-31.0, 61.5)	0.518	28.0 (-47.7, 103.7)	0.468
**L3 Erector Spinae Size (mm^2)**						
Baseline	1669.9 (404.6)		1723.2 (569.5)			
Δ 3 months	15.5 (-38.0, 69.1)	0.570	-15.8 (-63.7, 32.0)	0.517	31.0 (-41.2, 103.3)	0.400
Δ 6 months	32.7 (-20.8, 86.3)	0.231	22.9 (-26.1, 71.9)	0.360	9.5 (-63.6, 82.7)	0.799
**L4 Erector Spinae Size (mm^2)**						
Baseline	1283.1 (359.6)		1308.3 (465.2)			
Δ 3 months	20.4 (-15.4, 56.2)	0.265	-18.6 (-58.0, 20.9)	0.357	38.8 (-14.4, 91.9)	0.153
Δ 6 months	33.2 (-2.6, 69.0)	0.069	18.5 (-21.9, 59.0)	0.370	14.5 (-39.4, 68.3)	0.598
**L5 Erector Spinae Size (mm^2)[a]**						
Baseline	288.0 (310.9)		474.8 (372.1)			
Δ 3 months	37.9 (-11.1, 86.9)	0.801	-60.5 (-131.4, 10.5)	0.480	98.7 (14.0, 183.4)	0.709
Δ 6 months	-17.9 (-66.9, 31.0)	0.708	-39.0 (112.1, 34.1)	0.945	21.2 (-64.8, 107.2)	0.929

Data are: baseline unadjusted mean ± standard deviation (SD); within-group unadjusted mean absolute change with 95% confidence interval (CI); net difference (95% CI) were calculated by subtracting unadjusted within-group absolute changes from baseline to 3-and 6-months for MCMT and GSC.
[a]Underwent natural log transformation

TABLE S4. MRI outcomes for psoas major size of the middle three slices of each spinal level (L1-L5) at baseline, three and six months in participants randomized to general strength and conditioning (GSC) and motor control manual therapy (MCMT).

	**Baseline values and within group changes**				**GSC vs MCMT**	
	**GSC**		**MCMT**			
	**Mean (SD)<br>Mean (95% CI)**	**P-value**	**Mean (SD)<br>Mean (95% CI)**	**P-value**	**Net difference (95% CI)**	**Group x Time**
**L1 Psoas Major Size (mm^2)**						
Baseline	209.4 (168.1)		229.9 (153.9)			
Δ 3 months	3.9 (-20.1, 27.8)	0.750	-5.8 (-35.6, 23.9)	0.700	8.9 (-29.3, 47.1)	0.647
Δ 6 months	13.2 (-11.5, 37.9)	0.294	21.7 (-9.9, 53.5)	0.178	-9.1 (-49.2, 30.7)	0.654
**L2 Psoas Major Size (mm^2)**						
Baseline	563.2 (243.3)		601.2 (290.8)			
Δ 3 months	23.9 (-2.4, 50.2)	0.075	-2.3 (-24.2, 19,5)	0.836	26.5 (-8.0, 60.9)	0.132
Δ 6 months	12.1 (-14.2, 38.4)	0.366	2.5 (-19.9, 24.9)	0.828	9.9 (-25.0, 44.8)	0.577
**L3 Psoas Major Size (mm^2)**						
Baseline	1006.6 (291.0)		1001.2 (413.9)			
Δ 3 months	24.0 (-7.9, 55.9)	0.141	-11.8 (-34.0, 10.4)	0.296	36.0 (-3.4, 75.5)	0.073
Δ 6 months	22.9 (-9.1, 54.8)	0.160	-5.9 (-28.7, 16.8)	0.609	29.0 (-10.9, 69.0)	0.154
**L4 Psoas Major Size (mm^2)**						
Baseline	1359.9 (362.8)		1372.5 (464.5)			
Δ 3 months	26.8 (-7.2, 60.8)	0.122	-30.8 (-55.6, -6.0)	**0.015**	57.5 (14.9, 100.2)	**0.008**
Δ 6 months	29.1 (-4.9, 63.1)	0.093	2.0 (-23.4, 27.5)	0.875	27.0 (-16.2, 70.1)	0.221
**L5 Psoas Major Size (mm^2)**						
Baseline	1241.3 (384.3)		1203.8 (395.1)			
Δ 3 months	14.4 (-11.4, 40.2)	0.273	-11.9 (-38.4, 14.5)	0.377	26.3 (-10.6, 63.3)	0.162
Δ 6 months	8.4 (-17.4, 34.1)	0.525	9.6 (-17.5, 36.7)	0.487	-1.3 (-38.7, 36.1)	0.946

Data are: baseline unadjusted mean ± standard deviation (SD); within-group unadjusted mean absolute change with 95% confidence interval (CI); net difference (95% CI) were calculated by subtracting unadjusted within-group absolute changes from baseline to 3-and 6-months for MCMT and GSC.

TABLE S5. MRI outcomes for quadratus lumborum size of the middle three slices of each spinal level (L1-L5) at baseline, three and six months in participants randomized to general strength and conditioning (GSC) and motor control manual therapy (MCMT).

	Baseline values and within group changes				GSC vs MCMT	
	GSC		MCMT			
	Mean (SD)<br>Mean (95% CI)	P-value	Mean (SD)<br>Mean (95% CI)	P-value	Net difference<br>(95% CI)	Group x Time
**L1 Quadratus Lumborum Size (mm^2)**						
Baseline	195.8 (94.9)		188.9 (87.4)			
Δ 3 months	-0.6 (-21.1, 19.8)	0.950	-12.7 (-35.2, 9.8)	0.269	11.6 (-18.8, 42.0)	0.454
Δ 6 months	1.1 (-19.3, 21.5)	0.918	7.5 (-15.5, 30.6)	0.523	-7.1 (-37.9, 23.7)	0.651
**L2 Quadratus Lumborum Size (mm^2)**						
Baseline	290.4 (112.2)		303.3 (136.9)			
Δ 3 months	7.6 (-7.8, 23.1)	0.333	-8.7 (-25.3, 7.8)	0.302	16.3 (-6.3, 39.0)	0.157
Δ 6 months	-0.6 (-16.1, 14.9)	0.938	0.0 (-16.9, 17.0)	0.995	-0.7 (-23.6, 22.2)	0.953
**L3 Quadratus Lumborum Size (mm^2)**						
Baseline	416.0 (142.4)		446.5 (197.2)			
Δ 3 months	9.3 (-13.6, 32.3)	0.426	1.0 (-19.2, 21.3)	0.920	8.0 (-22.8, 38.8)	0.610
Δ 6 months	1.0 (-21.9, 23.9)	0.933	1.4 (-19.4, 22.1)	0.896	-0.7 (-31.8, 30.5)	0.967
**L4 Quadratus Lumborum Size (mm^2)**						
Baseline	497.2 (156.2)		512.5 (200.2)			
Δ 3 months	-16.6 (-43.7, 10.4)	0.228	-8.5 (-34.9, 17.9)	0.529	-9.2 (-47.2, 28.8)	0.636
Δ 6 months	10.2 (-16.8, 37.3)	0.458	10.3 (-16.9, 37.4)	0.458	-1.0 (-39.5, 37.6)	0.961

Data are: baseline unadjusted mean ± standard deviation (SD); within-group unadjusted mean absolute change with 95% confidence interval (CI); net difference (95% CI) were calculated by subtracting unadjusted within-group absolute changes from baseline to 3-and 6-months for MCMT and GSC.
